# Oxygen Generation Using Catalytic Nano/Micromotors

**DOI:** 10.3390/mi12101251

**Published:** 2021-10-15

**Authors:** Sumayyah Naeem, Farah Naeem, Jawayria Mujtaba, Ashish Kumar Shukla, Shirsendu Mitra, Gaoshan Huang, Larisa Gulina, Polina Rudakovskaya, Jizhai Cui, Valeri Tolstoy, Dmitry Gorin, Yongfeng Mei, Alexander A. Solovev, Krishna Kanti Dey

**Affiliations:** 1 Department of Materials Science, Fudan University, Shanghai 200433, China; sumayyahnaeem@gmail.com (S.N.); farah11naeem2013@gmail.com (F.N.); jawayria.m@icloud.com (J.M.); gshuang@fudan.edu.cn (G.H.); jzcui@fudan.edu.cn (J.C.); yfm@fudan.edu.cn (Y.M.); 2 State Key Laboratory for Modification of Chemical Fibers and Polymer Material Science and Engineering, Donghua University, Shanghai 201620, China; 3 Discipline of Physics, Indian Institute of Technology Gandhinagar, Palaj 382355, Gujarat, India; ashish.shukla@iitgn.ac.in (A.K.S.); shirsendu.m@iitgn.ac.in (S.M.); 4 Institute of Chemistry, Saint Petersburg State University, 26 Universitetskii Prospect, Petergof, 198504 St. Petersburg, Russia; l.gulina@spbu.ru (L.G.); v.tolstoy@spbu.ru (V.T.); 5 Center of Photonics & Quantum Materials, Skolkovo Institute of Science and Technology, 3 Nobelya Str., 121205 Moscow, Russia; p.rudakovskaya@skoltech.ru (P.R.); d.gorin@skoltech.ru (D.G.)

**Keywords:** micro-/nanomotor, oxygen, self-propulsion, hydrogen peroxide, catalysis, active matter

## Abstract

Gaseous oxygen plays a vital role in driving the metabolism of living organisms and has multiple agricultural, medical, and technological applications. Different methods have been discovered to produce oxygen, including plants, oxygen concentrators and catalytic reactions. However, many such approaches are relatively expensive, involve challenges, complexities in post-production processes or generate undesired reaction products. Catalytic oxygen generation using hydrogen peroxide is one of the simplest and cleanest methods to produce oxygen in the required quantities. Chemically powered micro/nanomotors, capable of self-propulsion in liquid media, offer convenient and economic platforms for on-the-fly generation of gaseous oxygen on demand. Micromotors have opened up opportunities for controlled oxygen generation and transport under complex conditions, critical medical diagnostics and therapy. Mobile oxygen micro-carriers help better understand the energy transduction efficiencies of micro/nanoscopic active matter by careful selection of catalytic materials, fuel compositions and concentrations, catalyst surface curvatures and catalytic particle size, which opens avenues for controllable oxygen release on the level of a single catalytic microreactor. This review discusses various micro/nanomotor systems capable of functioning as mobile oxygen generators while highlighting their features, efficiencies and application potentials in different fields.

Living organisms require a continuous supply of oxygen to drive their metabolic processes and sustain life. Cells use oxygen to convert the energy stored in foods, synthesize various proteins to form a healthy immune system and maintain muscles and organs [1,2]. Insufficient oxygen levels may lead to critical medical conditions such as chronic obstructive pulmonary disease, breathing difficulty and others [3,4,5,6]. Recently, oxygen concentrators have played an essential role in treating COVID-19 patients under critical care [7,8,9]. Lungs provide body with oxygen—humans use only 5% of oxygen per inhalation [10,11]. An oxygen concentrator is a device that takes in atmospheric air and removes the nitrogen from it, leaving an oxygen-enriched gas (typically 90–95%) used explicitly by hypoxemic patients [12]. Large-scale oxygen generation systems also find applications in power plants and other industries. These oxygen generation systems typically rely on cryogenic distillation technologies to supply pure oxygen at high pressures [13].

Several approaches can be used to generate gaseous oxygen, including photosynthesis [14,15,16], water splitting [17], oxygen concentrators [18,19], biological methods [20,21] and inorganic catalysis (Figure 1) [22,23,24,25,26]. During photosynthesis, plants absorb sunlight, water and carbon dioxide molecules and convert them into biomass and oxygen [14,15,16] following the reaction: sunlight + *n*H_2_O + *n*CO_2_ → (CH_2_O)*_n_* + *n*O_2_. The water-splitting reaction leads to the generation of O_2_ and H_2_ (Figure 1c) according to: 2H_2_O → 2H_2_ + O_2_. Both photocatalytic water-splitting and photosynthesis are essential processes of sunlight-based energy conversion strategies. Similarly, oxygen concentrators are direct commercial oxygen production methods that can deliver a continuous ~90% pure oxygen stream in response to the rapid pressure-swing adsorption (RPSA) process characterized by short cycle time and high productivity (Figure 1b). Oxygen can also be obtained from various biocatalytic and enzymatic reactions by lowering the activation energy and decomposition of hydrogen peroxide (H_2_O_2_) into water (H_2_O) and O_2_ (Figure 1d) [19,20,21]. Recently, much attention has been given to oxygen generation based on single catalytic nano/microparticles, which can act as efficient heterogeneous micro-reactors integrated on the substrate or released in solution, i.e., for “on-the-fly” oxygen delivery. In this regard, our understanding of the subtleties of catalytic decomposition of H_2_O_2_ and its dependence on the catalyst activity, surface area and reaction-diffusion processes are key parameters to improve for efficient oxygen generators [22,23]. H_2_O_2_ is known to be a stable source of oxygen in the presence of catalysts such as Pt, Ag, Pd, etc., following the stoichiometry: 2H_2_O_2_ → 2H_2_O + O_2_ [24,25,26]. The nature of the catalytic surface often plays a crucial role, setting the oxygen generation efficiency. For example, microtubular nanomembranes consisting of strain-engineered catalytic layers produce oxygen from hydrogen peroxide in the form of microbubbles. Tuneable catalytic microtubes with a high aspect ratio (length/diameter) are 1000 times more efficient than planar Pt catalytic surfaces [27].

This review is focused on general oxygen production methods and oxygen generation in nano/micromotors. To understand more about nano/micromotor fabrication, mechanisms of motion and potential applications, we refer readers to recently published excellent reviews [28,29,30,31,32]. This review discusses different methods to produce O_2_, followed by consideration of materials’ chemistry and other factors such as the fuel composition, size, shape, surface curvature and surface area of the catalyst for optimal oxygen generation based on an individual nano/microreactor. The correlation of self-propelled micromotors’ speed with gas evolution and a relevant study concerning drag and motive forces are discussed. Moreover, the amount of oxygen evolved from bubbling and non-bubbling surfaces of micromotors is estimated. In a recent study [33], the one-dimensional reaction-diffusion equation has been used to explain the possible mechanisms of mass transport and gas formation within the microtubes. Notably, the oxygen production amounts predicted by the gas generation equation match well with the experimental values. This review highlights different methodologies of oxygen generation and discusses the possible use of catalytic micro/nanomotors as motile gas generators. Oxygen generation can be achieved by standard chemical decomposition of several storage materials. These reactions are carried out in special chemical oxygen generators (COGs), in which chlorates, perchlorates or sometimes superoxides are decomposed (Figure 2). Some of the most widely used decomposition reactions are discussed below.

**Thermal decomposition of chlorates and perchlorates (candle chemistry)**: At very high temperatures (above 400 °C), sodium chlorates (NaClO_3_) and sodium perchlorates (NaClO_4_) decompose exothermally to produce gaseous oxygen and sodium chloride (NaCl). During the process, the temperature in the experimental chamber is maintained by the heat generated during the decomposition reaction. Notably, the reaction is highly exothermic, and temperature within the reaction chamber could often be as high as 600 °C, posing challenges to use it for general domestic or clinical purposes. In COGs, gaseous oxygen is produced using a mixture of sodium chlorate, catalyst, chlorine-suppressing agents, fuels, and binders. Transition metal oxides, for example, Co_3_O_4_, are typically used as catalysts in COGs. The fuels in these devices are supposed to facilitate continuous combustion. Interestingly, metal fuels such as cobalt, nickel and iron exhibit a certain degree of catalytic activity during the decomposition of sodium chlorate, which hardly influences the COG combustion characteristics. The main reaction of oxygen production in such devices is 2NaClO_3_ → 2NaCl + 3O_2_. However, part of the sodium chlorate can be decomposed by the reaction 2NaClO_3_ → Na_2_O + Cl_2_ + 5/2O_2_. To avoid chlorine accumulation, additives of a chlorine suppressor, such as Ba(OH)_2_, are used. Recently, a multilayer generator with different ratios of components in the layers has been developed to control the rate of oxygen formation [34]. COGs have been successfully used in aviation and submarines, but the high rate of oxygen formation and the high thermal effect of the reactions limit such generators’ routine applicability.

**Decomposition of metal peroxides**: Potassium superoxide (KO_2_) interacts with H_2_O to produce hydroxide and O_2_ in the process—4KO_2_ + 2H_2_O → 4KOH + 3O_2_; KOH +CO_2_ → KHCO_3_. The formation of KOH along with gaseous O_2_ finds important usage in developing rescue breathing systems in mines. KOH absorbs the CO_2_ in the exhaled air (as shown in the 2nd reaction), which at the same time gets replenished with fresh O_2_—facilitating breathing. Oxygen supply systems based on potassium superoxide decomposition show much promise in developing efficient on-demand oxygen delivery devices. In a study by Wang et al. [23], the performance of a potassium superoxide plate, operating under normal convection, was investigated. It was shown that increasing the air temperature and humidity could enhance the rate of oxygen generation. However, it might reduce the respiratory quotient (ratio of carbon dioxide produced to oxygen consumed by the body) due to the increased internal diffusion resistance. It was suggested that either using a higher number of superoxide plates or integrating the plates with a CO_2_ absorbing system might lead to a better oxygen generation rate and a higher value of the respiratory quotient. With the help of experimental data, a model was proposed to predict the oxygen generation rate, CO_2_ absorption rate, respiratory quotient and overall thermal output of the system. Furthermore, researchers have also investigated the oxygen generation experimentally on single plate-like potassium superoxide (PLPS) and developed a kinetic reaction model of the process based on the experimental data. A common disadvantage of using alkali metal peroxides as oxygen sources is the risk of explosion since the reaction is highly exothermic and requires an additional cooling circuit. Another challenge of using these reactions is the harmful effects that the produced alkali has on the environment.

**Decomposition of percarbonates**: Sodium percarbonate (2Na_2_CO_3_·3H_2_O_2_; a solid adduct of sodium carbonate and hydrogen peroxide) upon interacting with water, produces sodium carbonate and hydrogen peroxide: 2Na_2_CO_3_·3H_2_O_2_ → 2Na_2_CO_3_ + 3H_2_O_2_. In the presence of catalysts, the released H_2_O_2_ can be further decomposed to produce molecular oxygen at the desired rate. The advantage of using sodium percarbonate as an H_2_O_2_ source, even at higher temperatures, is due to its resistance towards auto-oxidation and degradation, unlike H_2_O_2_ without any stabilizers. Notably, the generation of oxygen from sodium percarbonate also produces foams, which may be used as detergents. However, the foams must be carefully removed if the generated oxygen is to be used for breathing purposes. This can be achieved using a mechanical foam breaker as commercially available antifoaming agents contain volatile organics and may pose additional challenges if mixed with oxygen [1].

A readily available oxygen supply at high concentrations can help provide primary health care to patients before the arrival of professional medical assistance. There are many devices for portable powdered oxygen delivery, such as EmOx, produced by Green Dot Systems, Inc. (Miami, FL, USA) [35], or SysO_2_, produced by System O_2_ Inc. (Roswell, GA, USA) [36]. These systems generate breathable oxygen through controlled chemical reactions and usually involve a reaction chamber that needs to be filled with tap water. The contents of two containers are then added to the reaction chamber. The container labeled “white powder bottle” contains sodium percarbonate, and the other container labeled “black powder bottle” contains a proprietary catalyst compound. For example, the EmOx system uses a manganese compound as a catalyst. A comparative analysis of tissue oxygenation was performed using different oxygen delivery systems and flow rates. The portable oxygen delivery devices usually have variable activation times and relatively low oxygen flow rates, making them less valuable for conditions where a significant oxygen supply is needed quickly. Oxygen concentrator systems, which provide a safe source of oxygen-enriched air, have been more promising under such emergency conditions than chemical oxygen generators. On the other hand, the low weight (up to 1 kg) and the absence of any power source or batteries make chemical oxygen generation devices indispensable in hard-to-reach conditions.

Percarbamide peroxide (CH_6_N_2_O_3_) is another solid source of peroxide, which consists of hydrogen peroxide compounded with urea and can deliver up to 17.0 weight % O_2_. Using percarbamide containing chitosan-coated hollow mesoporous silica nanoparticles, Huang et al. [35] developed an efficient free radical generation and delivery protocol, which is expected to find critical applications in cancer therapy. Typical disadvantages of solid percarbonates are poor control over the peroxide decomposition reaction, overheating the device structure, foaming and poisoning of the catalyst, which causes the reaction to stop unexpectedly. To overcome this difficulty, it has been proposed to press slowly on dissolving tablets of solid urea percarbonate (UHP) to control the rate of oxygen release [1]. Dissolution of urea is an endothermic reaction that balances the oxygen release reaction’s cumulative exothermic effect. In a study reported by Dingley et al. [33], the use of sodium percarbonate in the combined form of granules and slower dissolution tablets was recognized as the best option for the controlled generation of oxygen. The optimum combination ratio of sodium percarbonate, water and manganese dioxide catalyst was also calculated [37].

**Decomposition of H_2_O_2_ (catalytic disproportionation)**: Hydrogen peroxide is the best-known source of readily available molecular oxygen. H_2_O_2_ decomposes exothermally into water and oxygen: 2H_2_O_2_ → 2H_2_O + O_2_, and plays a crucial role in the functioning of hydrogen fuel cells [38], micro-pumps [39], motility of micro/nanomachines [40,41,42,43] within liquids, portable oxygen generators [44], decomposition of organic compounds in wastewater treatment [45], antiseptic treatment of fabrics [46], jet engines with hydrogen peroxide fuel [47] and metabolism of living cells [48]. The features of such reactions are given in a relatively large number of reviews, including those published over the last three years [49,50,51,52]. In considering each of these applications, various aspects of the catalytic disproportionation reaction of H_2_O_2_ are described. For example, the catalytic decomposition of 80–98% H_2_O_2_ is widely used in rocketry. The content of active oxygen depends on the concentration of hydrogen peroxide. When the concentration of H_2_O_2_ increases from 85 to 98%, the proportion of active oxygen changes from 40.0 to 46.1%, respectively, and the decomposition temperature increases from 907 to 1125 K in the same concentration range. The general requirements for a peroxide decomposition catalyst during the operation of such engines are detailed in a recent review [53]. The study highlights the minimum reaction start time after hydrogen peroxide makes contact with the catalyst, the high chemical stability of the process, the thermal and mechanical stability of the catalyst, among others. For example, the active phase, an expensive metal catalyst, is deposited as a thin-layer, highly porous support to increase the specific surface area. Use of platinum [54] or manganese oxide [55] deposited over aluminum oxide, structured networks of silver [56], granules and tablets of aluminum oxide with deposited layers of platinum or manganese oxide [57], cellular and honeycomb monoliths [58] from manganese oxides dispersed on monolithic zirconia substrates have been demonstrated in various studies for efficient oxygen generation. An essential characteristic of the catalyst which allows regulation of its functional properties is porosity or, more precisely, the ratio between specific surface area and mechanical strength. Thus, slow or incomplete decomposition of peroxide is possible on catalysts with a low specific surface area, while thermal effects can destroy a highly porous catalyst. In recent years, it has been proposed to use heat-resistant metal oxides as substrates to deposit catalysts. For example, in yttria-stabilized zirconia, ceramic honeycombs with manganese oxides as the active phase have been reported to have decomposition efficiencies exceeding 90% [59]. Similarly, the performance of lanthanum doped alumina catalyst supports was investigated in a high concentration hydrogen peroxide monopropellant thruster [60]. Silica-doped alumina-supported catalyst containing manganese oxide and cobalt oxide has been reported to be an ideal active phase combination for the decomposition of 98% peroxide [47]. Notably, the exothermic effect of the decomposition reaction of 50% hydrogen peroxide is used to launch underwater vehicles [61,62]. In this regard, the most effective catalysts are metals of the platinum group, Ag, Co, permanganates of alkali and alkaline-earth metals [63], manganese oxides, copper and chromium [64,65,66].

The chemical reactions of H_2_O_2_ decomposition on the surface of heterogeneous catalysts have been studied for a long time and continue to be the focus of modern research [67]. For example, decomposition of H_2_O_2_ on the Pt surface was reported in [68]. This study investigated the reaction mechanism by performing the reaction under different conditions, with particles of varied sizes and compositions. It was suggested that the decomposition of H_2_O_2_ over the surface of Pt nanoparticles occurs via a cyclic mechanism. Initially, one molecule of H_2_O_2_ reacts with the Pt surface to form PtO. Another molecule of H_2_O_2_ then reduces PtO to metallic Pt, releasing a molecule of H_2_O and O_2_. Particles that contain more PtO at their surface facilitate enhanced H_2_O_2_ decomposition. Furthermore, due to their lower work function, larger Pt nanoparticles also help in the rapid decomposition of peroxide. In a similar study, adsorption of H_2_O_2_ was studied over the surfaces of Au_10_ metal clusters and hydroxylated rutile TiO_2_ (110). The computational study suggested that H_2_O_2_ is likely to easily undergo HO-OH cleavage over Au_10_ clusters, forming hydroxyl groups, which eventually react to form water and oxygen. Plauck et al. studied the mechanism and nature of active sites responsible for the catalytic decomposition of H_2_O_2_ over Pd (111) and OH partially covered Pd (100) surfaces. The DFT calculations indicated that the reaction involves an O-O bond scission step, which is sensitive to Pd surface structure and largely governs the H_2_O_2_ decomposition activity. In ref. [69], Pd nanoparticles (PdNPs) on the surface of graphdiyne (GDY) nanosheets were studied, and it was shown that the O-O bonds in H_2_O_2_ molecules are weakened after adsorption on the surface of Pd nanoparticles and produce highly active free radicals, including OH˙ and OOH˙. Oxygen atoms in these radicals have a higher electronegativity than Pd atoms, and therefore, the oxidation state of the latter increases. However, when free radicals are converted into products, such as H_2_O and O_2_, they detach from the surface of PdNPs, and then the Pd atoms recover to their original state. It should be specially noted that the authors of this work show that PdNPs/GDY also performed as an oxygen generator to attenuate tumor hypoxia in a long-term therapeutic process in vivo. In another mechanism [70], Fenton’s reaction serves as an ideal route for oxygen generation while powering catalytic micromotor propulsion in solution.

Manganese oxide is widely used in the heterogeneous decomposition of H_2_O_2_ due to its excellent catalytic efficiency. It is usually used in powder due to its very high contact surface [71]. It has been reported that for manganese (IV) oxide with a pyrolusite crystal structure and a solution pH of 7, the observed decomposition reaction includes 15 different stages, and a pseudo-first-order rate model can represent the reaction kinetics. An important parameter that determines the reaction kinetics is the ratio [H_2_O_2_]/[MnO_2_]. It was shown that the hydroperoxide/superoxide anion is mainly formed as intermediates at a [H_2_O_2_]/[MnO_2_] ratio from 11.8 to 39.2. The maximum rate of decomposition of H_2_O_2_ and oxygen formation was reached when the concentration ratio was 11.8. It should be noted that the decomposition rate also increases with increasing pH. The decomposition of H_2_O_2_ molecules in a gas mixture on a manganese (IV) oxide surface is considered in [64]. Thin films of alkaline earth metal oxides, for example, a MgO film on the surface of a molybdenum substrate, can also catalyze the decomposition of H_2_O_2_. However, it is known that MgO block crystals do not catalyze such a reaction. Based on the studies performed with bare magnesia, extended bare magnesia and metal-supported magnesia, it was concluded that the nature of the substrate could significantly influence the dissociation and reduction of H_2_O_2_. The dissociative adsorption energy and hence the chemisorption strength was found to be dependent on the type of metal and thickness of the oxide layer over it. The enhanced reactivity of H_2_O_2_ over metal-supported oxide films was analyzed considering the structure, density of states, electron localization function, differential charge densities and specific occupied orbitals of the substrate. Moreover, the effect of solvent molecules present on the substrate surface over H_2_O_2_ decomposition was studied by characterizing the interaction of peroxide with magnesia thin films. A comparison of noble metals’ catalytic activity and a number of oxides is carried out in the paper [66]. It is shown that the first-order decomposition kinetics observed for Ag, Pt and MnO_x_ active phases and Ir and Pt–Sn alloy show zero-order kinetics. The morphology of the catalyst influences the catalytic activity—powdered samples have a higher catalytic activity than tablets. For samples with similar morphology, the increasing activity order is the following: Pt–Sn/Al_2_O_3_ < Ir/Al_2_O_3_ < Pt/SiO_2_ < MnO_x_/Al_2_O_3_ < Ag/Al_2_O_3_.

**Features of oxygen bubbles formed during the catalytic decomposition of H_2_O_2_**: The formation of O_2_ bubbles over an Au surface during catalytic decomposition of peroxide has been extensively studied. In a combined experimental and theoretical investigation, it was suggested that both catalysis and mass transport are responsible for the dynamic growth and evolution of the bubble. Under limited diffusion conditions, the growth of the bubble was found to be primarily dominated by gas diffusion rather than catalysis—which is expected to be valid for other catalytic surfaces as well. The diffusion-dominated bubble generation is ideal for realizing controlled gas evolution applications, such as the propulsion of self-powered motors in liquids. Furthermore, the delicate balance between O_2_ gas generation and diffusive gas transport may lead to well-repeated bubble growth behavior, which is likely to find many important applications in soft condensed matter physics. The morphology of the catalyst surface also plays a vital role in the formation of bubbles on its surface [72,73,74,75,76,77,78]. The influence of solid surface morphology on the growth of bubbles has been widely discussed in the literature, which finds importance in the optimization of processes involving heat exchangers, boilers, froth floatation, electrolytic decomposition of solutions and many other biomedical and technological applications [79,80,81,82]. There are strategies to fabricate superaerophilic and superaerophobic surfaces via synergic modification of surface topography and surface chemistry that may have important implications in molecular oxygen generation methodologies [83,84]. Preferential bubble formation, adsorption and ultrasonic cavitation on the hydrophobic part of a patterned surface prepared by micro-contact printing have also been demonstrated [85,86,87]. Using high-speed camera microscopy, it was further shown that hydrophobic surfaces of patterns interact predominantly with air bubbles [88]. These results were also highlighted in previous reports, where the impact of surface cavitation strongly depends on the substrate’s surface energy in a low-frequency ultrasound treatment [85,86,87]. In this study, an analysis of the bubble formation and cavitation on the pattern surface was carried out. The preferable interaction of gas bubbles, induced by both low and high-frequency ultrasound, with the hydrophobic part of the pattern surface was shown in [85,86,87].

One of the most crucial aspects to consider in the process of oxygen bubble generation through catalysis is creating a superaerophobic catalyst surface. Possible options for the formation of gas bubbles at the interface between a solid and a solution with a dissolved gas can be schematically described, as shown in Figure 3c. The catalyst’s surface should have irregularities and “peaks”, which could facilitate the nucleation of gas bubbles. The formation of the first bubble from the dissolved gas, its growth, and removal from the surface occurs precisely over these “peaks”. However, the merging of bubbles into larger ones over these “peaks” occurs to a lesser extent. Therefore, the sizes of such bubbles are smaller than the sizes of bubbles released under similar conditions on the surface of flat substrates. This is because they are formed at a lower supersaturation of the dissolved gas concentration.

Examples of superaerophobic samples are shown in Figure 3d,e. These include oriented arrays of 2D crystals (Figure 3d) and end surfaces of microtubes (Figure 3e). It was shown in [44] that the use of microtubes with Fe/Cr/Pt walls, and a length of 0.04 to 1 mm as a catalyst for the decomposition of hydrogen peroxide, makes it possible to obtain oxygen from solutions with a concentration of peroxide about 1000 times lower than that required for catalysts in the dispersed phase. Decomposition of H_2_O_2_ and the corresponding generation of oxygen have been extensively used to realize self-propelled systems at the micro and nanoscale. As mentioned earlier, the autonomous propulsion of rolled-up microtubes was demonstrated by Mei and Solovev, where the motors behaved as tubular gas-collecting cavities [33,39]. To understand the bubble nucleation, growth and recoil mechanism at the microscale, microtubes were immersed in hydrogen peroxide, resulting in the decomposition of the latter into water and oxygen at the catalyst surface. Bubble nucleation is typically observed on surface microcavities or defects as an outcome of gas supersaturation at the solid-liquid interface. The formation of bubbles over smooth surfaces (in the absence of cavities), on the other hand, involves higher levels of gas saturation. When surface cavities are present, the nucleation energy barrier is reduced, and less interfacial free energy is required to grow the bubbles.

At low levels of gas saturation, bubbles can only nucleate in solid cavities [91]. The Laplace equation is used to describe the pressure variation in a gas cavity, which can lead to the critical radius of bubble curvature [92], which is given by Δp/2γ where Δp represents the difference of pressure across the fluid interface and γ represents the surface tension. Microbubble growth occurs because of the decomposition of hydrogen peroxide catalytically without surfactant from the Ti/Cr/Pt rolled-up catalytic microtubes (Figure 4a). The bubbles may grow without recoil and could be a few hundred micrometers without imparting any motion to the micromotors. Supersaturation of the microtubular cavity with gaseous oxygen leads to unidirectional and bidirectional bubble recoils resulting in micromotor propulsion—the speed of which varies depending upon the concentration of chemical fuel and aspect ratio of the tubes [31].

The addition of surfactants plays a significant role in generating microbubbles, decreasing the surface tension and bubble size, stabilizing the air-liquid interface and increasing the bubble ejection frequency in catalytic microtubes [95]. Microbubble stabilization by a surfactant was first introduced by Felix Sebba [96]. The stabilization and micellar aggregation process after surfactant addition, and the decreasing effect of surface tension, are represented by the plot of surface tension versus surfactant concentration in Figure 4b. Firstly, below the CMC, surfactant molecules are separately present in bulk (monomers) and have higher surface tension at a low surfactant concentration. Secondly, the formation of the micelles (micellization process) occurs when the surfactant’s concentration in the solution increases and is above a critical micellar concentration (CMC). Thirdly, above the CMC, the surfactant molecules self-assemble to form micelles dispersed in the liquid, forming a colloidal suspension. At this point, the continuous decrease occurs in the interfacial tension but more slowly. The structure of the most commonly used anionic (sodium dodecyl sulfate (SDS)) and cationic (benzalkonium chloride (BCl)) surfactants are described in Figure 4c, left image. The microbubble’s size is dramatically changed after surfactant addition when the 50 µm long microtubes is immersed in the solution consisting of hydrogen peroxide and the surfactant. Therefore, due to the decrease of interfacial energy, interfacial tension decreases at the higher concentration of surfactants and helps in the generation and detachment of bubbles [92]. Besides the catalytic particle size, bubble generation is also dependent on the geometry of the motors. The bubbles can propel larger Janus catalytic micromotors, but Janus particles with a size less than 10 μm cannot be put into motion that easily [97]. The accumulated O_2_ requires critical energy for bubble nucleation on the surface of a solid in an aqueous environment. This critical energy, required for the bubble nucleation, is termed heterogeneous nucleation energy and relies on the saturation concentration of the O_2_ and the surface curvature. Catalytic surface curvatures such as concave, convex and flat directly influence the bubble nucleation energy and growth [98]. Figure 4d shows that the bubble nucleation on the concave surface requires less nucleation energy than the flat and convex surfaces. Ti-based catalytic nanoshell motors are fabricated to observe the bubble generation process, as shown in Figure 4e. Nanoshell motors can generate smaller-sized bubbles more easily compared to Janus motors. Methacrylic anhydride-based hydrogel micromotors consisting of hydrogel Janus capsules with e-beam-evaporated Pt catalyst (Pt-on-Caps) are fabricated to observe their self-propulsion. The individual Pt-on-cap micromotor self-propels itself by forming long O_2_ bubble patterns, as shown in Figure 4f. These micromotors can generate a sufficient motive force to carry a potential cargo [94]. Unidirectional microbubble generation from the catalytic microtube is represented in Figure 4g. In the presence of a catalyst, a significant amount of O_2_ can be released by the microtube following the disproportionation of H_2_O_2_ [33]. Manjare and co-workers suggested a simplistic reaction-diffusion equation that explains the mass transport and reaction within a tubular microjet [99]. The point of the maximum, where the O_2_ concentration is the highest, can be described by:

$$x_{\max}=\frac{1}{\beta }\sinh^{-1}\left(\frac{1}{\beta L}\left(1-\cosh\left(\beta L\right)\right)\right)+\frac{L}{2}$$

where, $\beta ={\left(\frac{2K}{D_{H_{2}O_{2}}R}\right)}^{\frac{1}{2}}$.

Here *K* is the reaction rate constant, $D_{H_{2}O_{2}}=1.43\times 10^{-9}m^{2}s^{-1}$ is the hydrogen peroxide diffusion constant, L and *R* are the length and radius of the tube, respectively. The reaction rate constant was formerly calculated by Paxton et al. as *K* = 6.83 × 10^−7^ m s^−1^ [99]. The generation of O_2_ has not been found to be symmetric along the tube, and for longer tubes, the position *x*_max_ shifts from midway to the non-bubbling end. Using tubular length and radius, the concentration of hydrogen peroxide fuel, and estimated beta value, the total produced oxygen mass’s theoretical value can be found to be nearly 10^−13^–10^−14^ kg s^−1^, which matches quite well with the experimental results [33]. The pressure gradient ∆P required to pump fluid along the tube length is ∆P = 12*v*μ/*d*^2^, where *v* is the average fluid velocity, *μ* is the fluid viscosity, and *d* is the tube diameter. Assuming the tube is 10 µm in diameter, the pressure value has been found to be 2 × 10^8^ Pa/m. It is hard to achieve a pressure-driven flow as it requires a significant external force. However, bubbles growing under pressure within a microtube experience an imbalance in interfacial forces and induce fluid pumping during its recoil. Resistance to laminar flow in the microtube can be estimated by considering the Hagen-Poiseuille flow as Navier-Stokes equations as in [100]. *R* = 8*μL*/π*r*^4^, where *μ* is the viscosity of the fluid, *L* is the length and *r* is the radius of the channel. Since the flow resistance depends on the fourth fold of diameter, even a minor change in the microtube diameter, i.e., conical shape, provides a highly unidirectional bubble recoil and fluid flow. However, when higher concentrations of chemical fuels are used, bubbles from both ends of the microtube are generated.

Understanding the kinetics and exact mechanisms of H_2_O_2_ decomposition over catalyst surfaces at different temperatures needs further careful investigation. The increasing demand for pure molecular oxygen has been augmented recently by the alarming environmental pollution and industrialization. In emergencies, portable oxygen generators are preferred compared to larger scale, bulky, expensive technologies with operational challenges [44,101]. Catalytic microtubes/micropumps may offer an ideal platform to produce oxygen from the chemically decomposed hydrogen peroxide, which could ideally lead to microscopic oxygen generators and transporters capable of operating under biologically relevant conditions. Figure 5 shows data related to the generation of oxygen microbubbles using tunable Ti/Cr/Pd catalytic microtubes and highlights how concentrations of hydrogen peroxide, surfactant and aspect ratio of tubes could influence the generation of oxygen bubbles by catalytic motors. Activation of microtubes of different lengths in various concentrations of H_2_O_2_ is described in Figure 5a [102]. Microtubes can generate microbubbles with minimum concentrations of H_2_O_2_, thus offering an inexpensive and efficient on-demand oxygen generation methodology. It is demonstrated that longer tubes are activated at the lowest concentration of H_2_O_2_ and have the highest efficiency among all other tubes. As expected, longer tubes confine molecular oxygen better, leading to a gas supersaturation and favorable conditions for the bubble nucleation at minimum fuel concentrations [103]. Molecular oxygen diffuses fast enough and eventually competes to achieve the supersaturation of gaseous oxygen within shorter tubes [39]. A schematic image of on-chip microtubes submerged in the aqueous solution of SDS surfactant and H_2_O_2_ is shown in Figure 5b. The dependence of the gas generation rate on the concentration of surfactant for Ti/Cr/Pd tubes of different lengths (45, 60, 75 µm) with a constant concentration of H_2_O_2_ (10% *v*/*v*) is represented in Figure 5c–e. The maximum gas generation rate is obtained with a 2% surfactant concentration. These results show that shorter tubes generate more total oxygen (despite their smaller catalytic surface), whereas the longer tubes produce oxygen bubbles at higher rates at the same fuel concentration. The use of surfactant reduces the solution surface tension, which leads to higher frequencies of bubble generation. Moreover, it is seen that the neck probably attaches bubbles at the tubular opening, which in turn assists them to grow to larger volumes and demands less energy than the bubbles, which divide into smaller volumes within the microtube.

The hydrogen peroxide decomposition rate increases at higher temperatures in the presence of a palladium catalyst. Researchers have realized it in terms of enhanced propulsion of 45 µm long catalytic microtubes at higher temperatures when put in H_2_O_2_ solution mixed with surfactants. Sanchez and co-workers achieved the super-fast motion of catalytic microtubes by merely increasing the hydrogen peroxide solution’s temperature [104]. Higher temperatures enable oxygen production at lower concentrations of hydrogen peroxide, facilitating more proficient bubble nucleation and growth. It is possible to generate significantly higher volumes of oxygen by a relatively smaller increase in the solution temperature. Typically, an increase in temperature increases the reaction rate owing to more collisions between the molecules of hydrogen peroxide and the catalyst surface, as it possesses sufficient activation energy for the desired disproportionation reaction [102]. This study provides a detailed understanding of how catalytic tubular microreactors in peroxide fuel set the oxygen evolution rates. It was also demonstrated how an external magnetic field could change the flow regime from turbulent to laminar transition metal dichalcogenide (TMD) micromotors. Oxygen generation is increased due to the higher residence time of the fuel over the surface of the catalyst [105].

Bowl-shaped nanometer-sized polymer stomatocytes with stable nanocavity were engineered for the storage of Pt-nanoparticles [106]. These stomatocytes, when introduced to a solution of aqueous H_2_O_2_, moved directionally with a rapid discharge of O_2_. The Pt-nanoparticles trapped in the cavity acted as catalysts to break H_2_O_2_ to form water and oxygen, thus creating a rapid gas discharge and imparting propulsion to the stomatocytes that behaved as “miniature monopropellant rocket engines”. Tu et al. [107] designed and studied the self-assembly of a similar type of hybrid stomatocyte nanomotor based on biodegradable poly (ε-caprolactone) (PCL), which encapsulated PtNPs, and decomposed H_2_O_2_ to molecular oxygen [106,108].

The propulsion mechanisms of nano/micromotors are determined not only by the size of the motors—which range from several nanometers [109,110,111] to several hundred micrometers [107,112,113,114,115]—but also by their functionalization and fabrication features. Nano/micromotors have been fabricated in many different shapes, including wires [115], tubes [27], rockets [116], stars [117], spirals [118] and spheres [94,119], which significantly influences their propulsion speed and directionality. An interesting system of polymer sphere, 3-methacryloxypropyl trimethoxysilane (TPM) that was designed to encapsulate a significantly higher portion of canted hematite cube, with part exposed to the fuel at pH 8.5—composed of 0.1 to 3% H_2_O_2_, 5 mM tetramethylammonium hydroxide, and 3.4 mM sodium dodecyl sulphate (SDS) was found to decompose H_2_O_2_ to molecular oxygen under illumination [120]. In this system, under blue light illumination, the exothermic decomposition of H_2_O_2_ is catalyzed by a hematite cube to form oxygen gas. Chemically generated O_2_ has also been harnessed for the operation of an oxygen micropump during the catalytic decomposition of hydrogen peroxide [121,122]. A light-controlled bubble-propelled metal oxide TiO_2_ tubular microengine with an average diameter of 6 µm and length of 50 µm was fabricated, in which the gaseous O_2_ molecules are generated from the photocatalytic H_2_O_2_ decomposition under UV illumination. The propulsion of the tubular microengine could be reversibly controlled by switching the UV light ON and OFF [123]. A micromotor based on TiO_2_ and Au could use pure water for simultaneous propulsion and evolution of oxygen gas [124]. This light-driven micromotor system of diameter close to 1 µm could achieve a propulsion speed of about 25 body lengths per second, which could be turned ON and OFF on demand by external UV illumination. When a low amount (~0.1%) of H_2_O_2_ was added, the propulsion speed of the motor increased significantly [125]. A Janus micromotor, consisting of black TiO_2_ (B-TiO_2_) and Au, displayed light-induced self-propulsion within a wide range of wavelengths due to good light absorbance of B-TiO_2_ together with the photocatalytic activity of TiO_2_ [126]. Chemically generated O_2_ gas has been observed with a TiO_2_-based asymmetric Janus micromotor in pure water with enhanced propulsion speed [127]. A low-cost inorganic metal oxide (MnO_2_) catalyst-based micromotor was fabricated that exhibited simultaneous O_2_ generation and self-destruction properties and could potentially be used for controlled drug release in solution [128].

Janus spherical nano/micromotors are very attractive for their ease of fabrication and functionality [113,129,130]. There are two main approaches—physical vapor deposition (PVD) [131], which can asymmetrically functionalize nano/microspheres with layers of catalysts or other molecules, and the chemical one. Based on PVD, angular deposition [132] can change the coating of the layers with different functional materials. Approaches based on microcontact make it possible to “stamp” nanoparticles (NPs) over particles [133]. Controlling the thickness of the protective layers is another strategy to endow Janus nano/micromotors with desired coverage of functional materials [134,135]. Other chemical routes are widely used to make Janus spheres, such as emulsion solvent evaporation [136], bipolar electrodeposition [137] and the Pickering emulsion method [138,139]. The study reported in [140] demonstrates an inexpensive and high-performance method for the synthesis of hydrogel microspheres with sizes ranging from 20 to 500 μm, based on the photochemical solidification of hydrogel followed by the attachment of functional nanoparticles, which yields uniform hydrogel microspheres coated with desired molecules or catalysts. The functionalization of the spheres is carried out by attaching various functional nanoparticles (MnO_2_, TiO_2_ and Fe_3_O_4_) to the microspheres that enable the motors to move in various media (pure water, H_2_O_2_ solution and methylene blue (MB) solution) under different influences (light and magnetic field). The method’s primary feature is its flexibility—the ability to load two types of functional nanoparticles (MnO_2_ and Fe_3_O_4_) onto hydrogel microspheres in a single and direct step. It is possible to incorporate chemical catalysts (MnO_2_) [114,119], photocatalysts (TiO_2_) [141,142,143] and magnetic materials (Fe_3_O_4_) [144] into the structure of Janus nano/micromotors. Janus multifunction micromotors operate efficiently in H_2_O_2_ solution but can also be controlled by external magnetic fields. The basic idea behind Janus multifunctional micromotors is to attach functional nanoparticles to microspheres using a photocurable hydrogel precursor. A crosslinked hydrogel, in which the nanoparticles are embedded within the polymer matrix, allows the fuel to penetrate and come in contact with the nanoparticles, thereby allowing faster bubble generation and enhanced propulsion speed. Another example showed an application of 2D nanomaterials (graphene oxide, graphdyine oxide, black-phosphorus) to fabricate Janus motors with integrated bubble, magnetic and light powered motive mechanisms [145]. Oscillatory motion in combination with light emission was demonstrated by alginate hydrogels with Prussian Blue. Oxygen bubbles were generated in contact with Prussian blue playing the role of catalyst, while a light emission was simultaneously achieved in the presence of luminol [146].

To increase the catalytic micromotors’ speed, or set their direction, additional functionalities (such as light-sensitive nanoparticles or magnetic nanoparticles in their structure) have been introduced. Magnetic coatings are commonly used to achieve directional control of micromotor motion, such as Fe_3_O_4_ NPs, Fe films [27], permalloy caps [147] and [Co/Pt(Pd)] multilayer bags [148,149]. It has been shown that Fe_3_O_4_ Janus microspheres are self-propelled in 30 wt% H_2_O_2_, where their trajectory is about four times longer than in pure water. Fe_3_O_4_ can decompose H_2_O_2_ in the same way as MnO_2_, acting as a catalyst. In the field of active matter, the magnetic properties of Fe_3_O_4_ nanoparticles in H_2_O_2_ are harnessed more often than their catalytic activity [150]. In [135], micromotors were created from a composite structured polymer with two separate cores of hydrogels containing Pt and dispersed Fe_3_O_4_. These micromotors are based on a dual internal injection microfluidic capillary system. The diameter of micromotors, the size and number of inner cores and the amount of Pt and Fe_3_O_4_ nanoparticles in the cores can be adjusted by controlling the flow rate of pre-polymerized solutions. Upon catalyzing H_2_O_2_ decomposition, the Pt-integrated cores generate sufficient propulsion force while Fe_3_O_4_ provides the necessary control to veer the motor towards specific directions within fluidic media and deliver colloidal cargo at desired locations. These results demonstrated that the microfluidic emulsification technique was helpful for the one-step generation of composite micromotors [151,152]. The speed of functionalized micromotors is 100–400 μm s^−1^; however, under a low-frequency magnetic field, samples containing magnetic components have a speed from ~204 μm s^−1^ to 1100 μm s^−1^. With the help of a magnetic core, we can control the propulsion of the micromotor, and at the same time, we can steer its propulsion in the desired direction.

Multifunctional self-propelled microrobots are often developed for biomedical applications [153,154,155]. Due to their self-propelled mechanism, they are the most effective carriers of drugs compared to microcapsules and nano-objects. The studies [141,154,156] report the fabrication of multifunctional superparamagnetic/catalytic microrobots for cell manipulation, loading of anticarcinogenic drugs and their delivery to cancer cells. The complex structure of microrobots leads to a triple functionality—a polymer layer rich in functional groups (tosyl, hydroxy, amino) can bind molecules and biological materials, a catalytic layer (platinum, manganese oxide, and other objects for catalyzing peroxide decomposition) ensures the movement of motors and the magnetic component enables motion control using external magnetic fields. The micro-robots described above can move one at a time and combine under the influence of a weak magnetic field, forming chains that allow the capture and transportation of storage cells. In the study [154], magnesium-based micromotors with the Janus structure were modified with iron oxide [70]; bonding occurs through electrostatic interactions. It was shown that when a magnetic field was applied, the speed of the micromotors increased by 13%, besides achieving controls over the directionality of propulsion. Furthermore, these micromotors had low cytotoxicity, and the viability of their cells, corresponding to 100 μg/mL, was almost 80%. It has been shown that micromotors containing drugs [15,141] are more effective than the drugs themselves in a static mode. Another example showed AuNW nanomotors loading, carrying and delivering vehicles for intracellular oxygen transport, which increase cell viability under hypoxic conditions [157].

Much like chemical reactions, biochemical transformations also offer some of the most common and economical routes for generating oxygen in solutions that may be used in a variety of specific applications at the micro/nanoscale. Sengupta et al. [158] reported an enhanced diffusion of catalase upon interaction with their substrate, i.e., in H_2_O_2_ solution. Using fluorescence correlation spectroscopy, it was observed that despite an ultra-small hydrodynamic radius of around 7 nm, movements of enzymes become directional in a fuel gradient. In other words, according to this scenario, enzymes become chemical motors that self-propel [158]. However, observed diffusion enhancement of enzymes is at least one order of magnitude higher than predicted theoretical results [159]. This is an area of recent debate and controversy in the literature. For example, Fischer’s group showed that fluorescence signals could suffer from artifacts. The absolute diffusion constants of enzymes were measured without chemical/optical labeling with Pulsed Field Gradient Nuclear Magnetic Resonance (PFG-NMR) [160]. In contrast, larger objects, such as micron-sized polystyrene particles, functionalized with catalase in H_2_O_2_ substrate, display enhanced propulsion [161]. The motion of these microbeads could even be directed by using steady substrate concentration gradients within a microfluidic environment. Such bio-hybrid systems also generate oxygen from peroxide fuel, concentrated using appropriate architectures and stored for future applications. An Au-SiO_2_-based nanomotor was synthesized, having dimensions nearly 800 nm, capable of generating gaseous O_2_ in an aqueous solution of H_2_O_2_, and H_2_ bubbles in the presence of NaBH_4_ [152]. These nanomotors are capable of decomposing H_2_O_2_ (due to the presence of Au nanoparticles) and have been shown to move with an average speed of 50 μm/s in 3% H_2_O_2_ solutions [162]. Mesoporous Janus nanomotors based on silica nanoparticles and catalase, of dimension nearly 400 nm, were synthesized by Ma et al. [120]. These nanomotors in 1.5% *w*/*w* H_2_O_2_ substrate catalyzed chemical reactions and generated oxygen molecules as a result. Molecular oxygen was produced due to catalase which is known to be extremely specific and efficient [163]. A similar kind of silica-based nanomotor, coated with a thin layer of Pt, was made for active drug delivery and produced O_2_ gas in the H_2_O_2_ substrate. The motors are of the dimension of 50 nm with 100% enhancement in their diffusion coefficient in solution compared to their Brownian motion [131]. Another bio-hybrid micromotor, of dimension ~10 µm, uses catalase enzymes functionalized over biotinylated polymer vesicles and converted H_2_O_2_ to water and oxygen gas, resulting in the motion of the vesicles. The catalyst in the presence of H_2_O_2_ created a differential impulsive force that resulted in the movement of the vesicles [164]. Catalase has also been used in powered motors and generating oxygen in systems designed for DNA detection [165] and water quality monitoring [166]. Pt-deposited nanomotors of dimension ~50 nm were fabricated using lower-dimensional nanomaterials, and H_2_O_2_ was used for their movement [167]. Due to the platinum coating on their surface, the motors decomposed H_2_O_2_ and generated oxygen gas in the process. Besides powering micro/nanoscopic particles, active enzyme molecules, when attached over a solid surface, have also been shown to produce oxygen and generate sufficient mechanical forces to cause directional fluid pumping [168,169,170]. The pumping speed increased with increasing substrate concentration and rate of enzymatic catalysis. The pumps were rechargeable and could be triggered by the presence of specific molecules, which opens avenues for the fabrication of biomolecule-powered smart sensors and on-demand oxygen delivery microsystems.

Previously, toxic peroxide fuel represented a significant issue for biomedical applications of nanomotors. However, significant progress has been achieved to solve this issue. Firstly, since catalytic nano/micromotors cannot overcome high blood flow speeds, the idea of in vivo micromotor motion induced by the catalytic reaction is questionable. Secondly, different kinds of biocompatible micromotors can be prepared using microfluidic methods, particularly designed for ultrasound imaging, diagnostics and therapy [171]. Thirdly, a proof-of-concept of applied selective and sequential catalytic nanomedicine for effective tumor treatment has been reported. For example, nanocatalysts (glucose oxidase, Fe_3_O_4_) are delivered to tumor sites using this approach. Glucose oxidase depletes glucose in the tumor and generates hydrogen peroxide. Subsequently, Fe_3_O_4_ catalyzes hydrogen peroxide (in an acidic tumor environment) and generates hydroxyl radicals, leading to apoptosis and deactivation of tumor cells [172]. Table 1 shows the influence of micromotor shape, size, material, concentration of hydrogen peroxide and composition of hydrogen peroxide on the volume of produced oxygen gas. Generally, increasing substrate concentration of hydrogen peroxide increases the rate of decomposition reaction. The addition of surfactants reduces hydrogen peroxide surface tension and leads to the enhancement of oxygen bubble evolution. However, it depends critically on the micromotors’ shape [39,98] and surfactant type [91], which are not yet fully understood.

In summary, during recent years, oxygen-generating nano/micromotors have been the subject of systematic investigation. Nano/micromotors are widely used for potential applications as nano/micromachines in diverse fields. Multiple micromotors convert hydrogen peroxide as their fuel to achieve motion, decomposing it into oxygen and water. Chemical micromotors include a catalytic segment that provides a motive force in various conditions and environments. The in situ and controllable production of gaseous oxygen is essential to realize multiple applications, including on the fly sensing, mass transport and delivery of molecules at desired locations under complex environments. Although much work has already been done on these fronts in active matter, the potential application of such systems towards oxygen generation and storage has not been investigated vigorously. On-the-fly generation of pure oxygen at the micro/nanoscopic level in gaseous form is likely to open up newer avenues in molecular transport and therapy. By controlling the structure, size, shape, methods of preparation, functionalization, aspect ratio of catalytic nano/microparticles and peroxide fuel concentration and composition, oxygen can be released on demand. Other key parameters to increase the generation of oxygen are the surface roughness and porosity of catalytic nano/microreactors. Choudhury and co-workers have reported the fabrication of platinum silica nanomotors with increased roughness [191]. Physical vapor deposition (PVD) was used to increase the surface area of nanomotors that subsequently led to a four-fold increase in velocity. Wang’s group reported self-propelled catalytic Pt-black/Ti Janus micromotors generating H_2_ bubbles in NaBH_4_ fuel [192]. The micromotor-based generation of energetically rich gas products was used for powering a small car prototype with a fuel-cell involving in situ H_2_ and O_2_ generation. In addition, the same Pt-black/Ti micromotors were used to release O_2_ (in H_2_O_2_ fuel) at separate volumes to power fuel-cell model cars. Mei’s group reported catalytic microtubes with hierarchical nanoporous walls [193]. In comparison to the smooth surface of microtubes, more efficient oxygen microbubble generation was observed in nanoporous tubes. Subsequently, this led to fast speeds of microtubes in hydrogen peroxide solution. Therefore, controllable and efficient generation of usable oxygen by micro and nanomotors may lead to the fabrication of multifunctional active materials with high potential for specific applications.

## Figures and Tables

**Figure 1:**
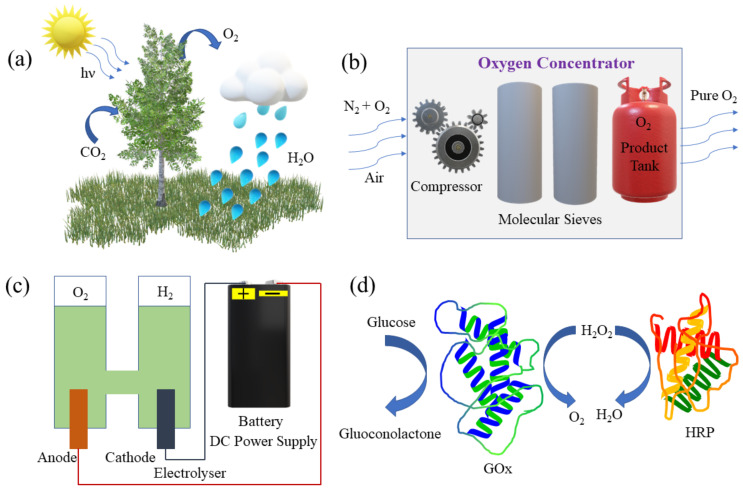
Schematic images of different oxygen generation methods. (**a**) Photosynthesis: plants convert carbon dioxide to produce oxygen. (**b**) A portable oxygen concentrator. (**c**) Water splitting reaction. (**d**) An example of enzymatic catalysis.

**Figure 2:**
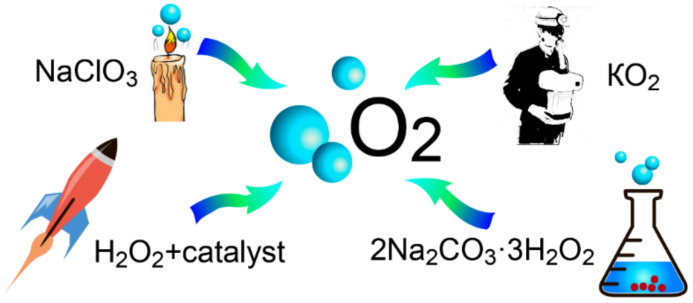
Oxygen production from storage materials via chemical reactions.

**Figure 3:**
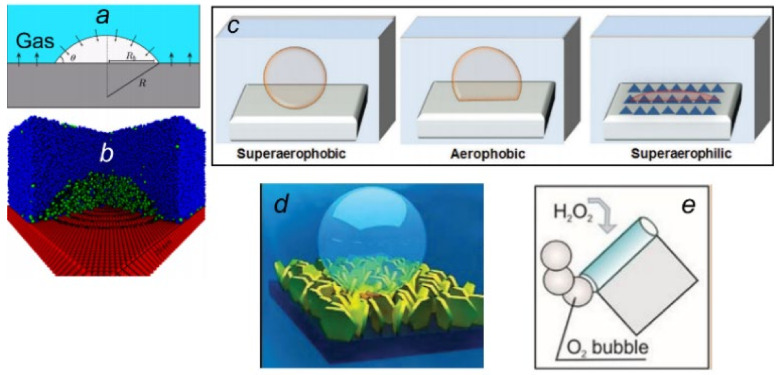
Schematic illustration of the formation of a gas bubble on a (**a**) flat and (**b**) rough surface. Reproduced with permission from Ref. [89], Copyright 2020, American Chemical Society. (**c**) Schemes for gas bubble formation on superaerophobic, aerophobic and superaerophilic surfaces. Reproduced with permission from Ref. [84], Copyright 2017, WILEY-VCH Verlag GmbH & Co. KGaA, Weinheim. Examples of catalysts with superaerophobic properties: (**d**) An array of 2D crystals. Reproduced with permission from Ref. [90], Copyright 2019, Elsevier. (**e**) Microtube faces. Reproduced with permission from Ref. [39], Copyright 2019, Wiley-VCH Verlag GmbH & Co. KGaA, Weinheim.

**Figure 4:**
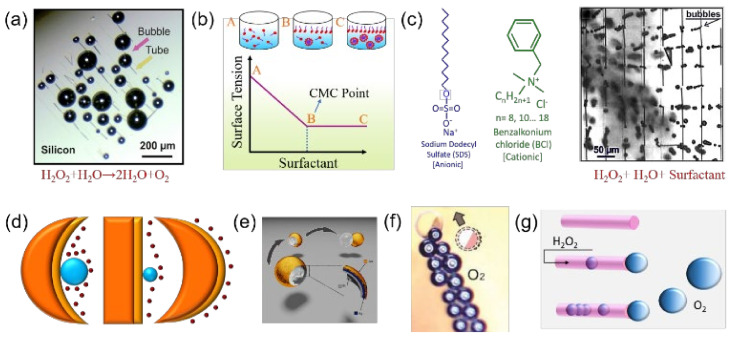
(**a**) Catalytic micromotors in an aqueous solution of H_2_O_2_ (without surfactant). Bubbles are indicated by the pink arrow, which grows due to the H_2_O_2_ decomposition into H_2_O and O_2_ because of the Pt catalyst inside the microtube (yellow arrow). The microbubble diameter was more than 100 µm and could not detach from the microtube. Reproduced with permission from Ref. [88]. (**b**) (Top image) Schematic process of bubble stabilization by surfactant absorption and formation of micelles. (Bottom image) Plot of the surface tension versus surfactant concentrations. Reproduced with permission from Ref. [88]. (**c**) Examples of different surfactant types (anionic and cationic). Effect of surfactant addition (common dish soap, “Fit-waschmittel”) on the reduction of bubble size. Reproduced with permission from Ref. [88]. (**d**) Schematic representation of bubble growth on the concave, flat and convex surfaces. (**e**) Schematic of bubble emission from the concave surface of nanoshell catalytic motors. Reproduced with permission from Ref. [93], Copyright 2013, American Chemical Society. (**f**) Optical micrograph of long tails of O_2_ bubbles produced by an individual Pt-on-Caps micromotor in H_2_O_2_ and dish soap solution. Reproduced with permission from Ref. [94], Copyright 2019, IOP Publishing Ltd. (**g**) Schematic illustration of oxygen bubble nucleation and recoiling from the catalytic microtube.

**Figure 5:**
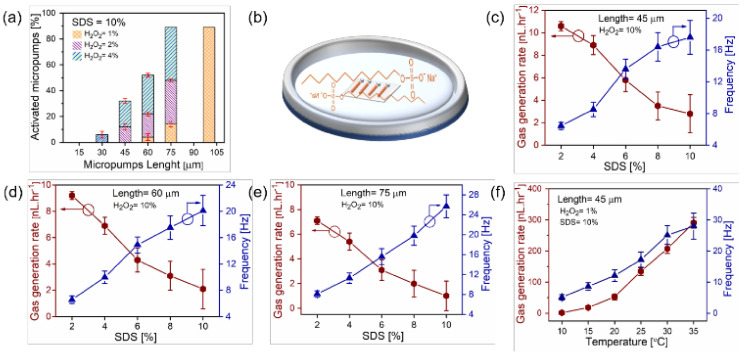
Activation of microtubes at a constant SDS (10% *v*/*v*) and different concentrations of H_2_O_2_, which are represented by the cross right-tilted and left-tilted regions. (**a**) Dependence of micropumps’ lengths on activity, i.e., generation of oxygen microbubbles. (**b**) A schematic image of the SDS molecule and catalytic microtubes in the H_2_O_2_ solution. (**c**–**e**) Analysis of oxygen gas generation rate and bubble frequency from the different microtube lengths at constant H_2_O_2_ concentration. (**f**) Oxygen generation from a 45 µm catalytic microtube at various temperatures. Reproduced with permission from Ref. [102], Copyright 2020, MDPI.

**Table 1:** Comparison of micro/nanomotor shape, size, material, hydrogen peroxide concentration, hydrogen peroxide composition and volume of generated oxygen.

Motor Shape	Motor Size	Motor Material	H_2_O_2_ Concentration	H_2_O_2_ Composition	Volume of O_2_ per sec	Ref.
Microparticles	35 μm	Ag	9%	9% H_2_O_2_ and 0.5% SDS	4.29 × 10^−3^ nL	[173]
Microparticles	5 μm	MnO_2_	12%	12% H_2_O_2_ and 0.5% SDS	1.21 × 10^−3^ nL	[173]
Rolled-up catalytic microtubes	20 μm-long tubes	Ti/Cr/Pt	6%	6% H_2_O_2_ and 32% soap	3.38 × 10^−3^ nL	[44]
Nanoparticle-shelled microbubbles	170 μm	Ti/Pt catalyst	14%	14% H_2_O_2_ and 10% PVA	5.144 nL	[174]
Catalytic Nanoshell Micromotors	2 μm	Pt–Ag–Au layers onto silica beads	5%	1.8% H_2_O_2_ inside the shell and 5% H_2_O_2_ outside the shell	4.39 × 10^−4^ nL	[93]
Catalytic microtubular engines	2 μm diameter, 8 μm long	PANI/Pt bilayer tube	10%	10% H_2_O_2_ and 1.6% NaCh	1.18 × 10^−3^ nL	[22]
Disk-like	8 μm diameter	Au-Ni-Pt	2%		3.35 × 10^−4^ nL	[175]
Janus heparin-loaded ammoniated-hollow mesoporous silica (H-A-HMS) nanomotor	250 nm	Fe_3_O_4_ core, CTAB mesoporous template agent, ammoniated process, Pt decorating onto the partial surface	15%	15% H_2_O_2_ and 0.3% SDS	6.93 × 10^−6^ nL	[176]
Hollow dumbbell-shaped	10 μm	MnO_2_	10%		1.15 × 10^−3^ nL	[177]
Disk-like micro-craft	12 μm diameter	Au–Ni–Pt	20%		1.18 × 10^−2^ nL	[178]
Janus nanoparticles	320 ± 20 nm	TiO_2_/MnO_2_	15%	10% H_2_O_2_ and 0.2% SDS	2.2 × 10^−6^ nL	[110]
ACFs (active carbon fibers)-based micromotors	15 μm	Mn_3_O_4_@ZnO/ACFs micromotors	7%	7% H_2_O_2_ and 1.25% SDS	2.3 × 10^−2^ nL	[179]
Metal sandwiched polytryptophan body	6 μm long, 400 nm diameter	Au/poly-Trp/Co	10%		20 nL	[180]
Spherical particle	10 µm	MnO_2_	5%	5% H_2_O_2_ and 0.1% SDS	3.1 × 10^−2^ nL	[181]
Pot-like hollow particle	25 µm	MnFe_2_O_4_/OA	2%	2% H_2_O_2_ and 0.1% CTAB	8.5 × 10^−2^ nL	[182]
Janus particle	5.6 µm	rGO/γ-Fe_2_O_3_/SiO_2_-Pt	10%	10% H_2_O_2_ and 1.5% NaCh	1.2 × 10^−2^ nL	[183]
Spherical particle	5 µm	Au/Pt/TiO_2_	10%	10% H_2_O_2_ and 1% SDS	1.7 × 10^−3^ nL	[184]
Janus, spiky	30 ± 0.4 µm	Sporopollenin exine capsules (SECs)	7%	7% H_2_O_2_ and 0.3% of SDS	4.2 × 10^−2^ nL	[185]
Janus, spherical	80 µm	Ag-ZIF	20%	20% H_2_O_2_ and 0.2% of SDS	5.7 nL	[186]
Microtubes	15 and 45 μm	Ti/Cr/Pd	8%	8% H_2_O_2_ and 10% SDS	8.1 × 10^−2^ nL	[187]
Cylindrical	3 mm diameter, 3 mm long	KMnO_4_/PAM hydrogel (H-motor)	10%		2.7 × 10^4^ nL	[112]
Tubular hydrogel	0.7 cm	PB/SDS/rGO/hydrogel	7.5%		6.6 × 10^3^ nL	[187]
Tubular hydrogel	0.7 cm	PB/SDS/rGO/hydrogel	22.5%		1.7 × 10^4^ nL	[187]
Cylindrical	1 mm diameter and 7 mm long	Enzyme/rich tissue	0.05%	0.05% H_2_O_2_ and 0.5% SDS	31 nL	[188]
Rods	5 μm diameter and 14 μm long	ZIF-8 rods loaded with Fe_3_O_4_ NPs and chemical deposition of Pt NPs	5%	5% H_2_O_2_ and 1% SDS	6.7 × 10^−3^ nL	[189]
Cylindrical rolled-up tubes	600 nm diameter and 10 μm long	InGaAs/GaAs/(Cr)Pt	20%	20% H_2_O_2_ and 10% soap	3.1 × 10^−5^ nL	[109]
Tube	4.6 µm	Pt/Ni/GOx	1.5%	1.5% H_2_O_2_ and 0.1% SDS	5.5 × 10^−3^ nL	[190]
